# Investigating cellular similarities and differences between upper tract urothelial carcinoma and bladder urothelial carcinoma using single-cell sequencing

**DOI:** 10.3389/fimmu.2024.1298087

**Published:** 2024-06-06

**Authors:** Qingyun Zhang, Chengbang Wang, Min Qin, Yu Ye, Yingxi Mo, Qinggui Meng, Guanglin Yang, Guanzheng Feng, Rui Lin, Shinan Xian, Jueling Wei, Shaohua Chen, Shan Wang, Zengnan Mo

**Affiliations:** ^1^ Department of Urology, Guangxi Medical University Cancer Hospital, Nanning, Guangxi, China; ^2^ Center for Genomic and Personalized Medicine, Guangxi Key Laboratory for Genomic and Personalized Medicine, Guangxi Collaborative Innovation Center for Genomic and Personalized Medicine, Guangxi Medical University, Nanning, Guangxi, China; ^3^ Human Sperm Bank, The First Affiliated Hospital of Guangxi Medical University, Nanning, China; ^4^ Department of Emergency, The Second Affiliated Hospital of Guangxi Medical University, Nanning, Guangxi, China; ^5^ Department of Research, Guangxi Medical University Cancer Hospital, Nanning, Guangxi, China

**Keywords:** tumor microenvironment, upper tract urothelial carcinoma, cellular heterogeneity, single-cell RNA sequencing, interaction network

## Abstract

**Background:**

Upper tract urothelial carcinoma (UTUC) and bladder urothelial carcinoma (BLCA) both originate from uroepithelial tissue, sharing remarkably similar clinical manifestations and therapeutic modalities. However, emerging evidence suggests that identical treatment regimens may lead to less favorable outcomes in UTUC compared to BLCA. Therefore, it is imperative to explore molecular processes of UTUC and identify biological differences between UTUC and BLCA.

**Methods:**

In this study, we performed a comprehensive analysis using single-cell RNA sequencing (scRNA-seq) on three UTUC cases and four normal ureteral tissues. These data were combined with publicly available datasets from previous BLCA studies and RNA sequencing (RNA-seq) data for both cancer types. This pooled analysis allowed us to delineate the transcriptional differences among distinct cell subsets within the microenvironment, thus identifying critical factors contributing to UTUC progression and phenotypic differences between UTUC and BLCA.

**Results:**

scRNA-seq analysis revealed seemingly similar but transcriptionally distinct cellular identities within the UTUC and BLCA ecosystems. Notably, we observed striking differences in acquired immunological landscapes and varied cellular functional phenotypes between these two cancers. In addition, we uncovered the immunomodulatory functions of vein endothelial cells (ECs) in UTUC, and intercellular network analysis demonstrated that fibroblasts play important roles in the microenvironment. Further intersection analysis showed that MARCKS promote UTUC progression, and immunohistochemistry (IHC) staining revealed that the diverse expression patterns of MARCKS in UTUC, BLCA and normal ureter tissues.

**Conclusion:**

This study expands our multidimensional understanding of the similarities and distinctions between UTUC and BLCA. Our findings lay the foundation for further investigations to develop diagnostic and therapeutic targets for UTUC.

## Introduction

Urothelial carcinoma (UC) is a highly aggressive genitourinary cancer that originates from the urothelial cells lining the urinary tract, including the bladder, ureters, and renal pelvis (1). Among these, upper tract urothelial carcinoma (UTUC) arises from the urothelium of the renal pelvis and ureter, and is known to be highly aggressive, has an insidious onset, high recurrence rates, and rapid progression (2). The prevalence of UTUC has been on the rise, accounting for approximately 10% of UC cases in Europe and the United States (3), with higher proportions in China, ranging from 9.3% to 29.9% of UC cases (4). Importantly, UTUC and bladder urothelial carcinoma (BLCA) both originate from uroepithelial tissue, presenting remarkably similar clinical manifestations and histological features. Consequently, the diagnostic and therapeutic approaches for UTUC are mainly derived from those of BLCA (5). However, emerging evidence suggests that even identical treatments may lead to poorer outcomes in UTUC compared to BLCA (6). The mainstream therapeutic strategy for advanced UTUC, platinum-based chemotherapy, is associated with undesirable side effects and has low overall response rates (7). Furthermore, the sensitivity of existing non-invasive diagnostic methods for UTUC are unsatisfactory, highlighting the urgent need for developing noninvasive and effective complementary diagnostic approach for this cancer. Research on UTUC and the underlying biological mechanisms driving its malignancy lags significantly behind. Moreover, knowledge of therapeutic targets specific to UTUC and its biological differences compared to BLCA remains insufficient, emphasizing the need for accelerated efforts to bridge these gaps and advance our understanding of treatment options.

The tumor microenvironment (TME) is a biological milieu that influences tumorigenesis and progression. It is shaped by interactions among tumor cells, immune cells, stromal cells, extracellular matrix (ECM), and secreted molecules (8). These complex orchestrations and interactions among diverse cell subpopulations within the TME profoundly influence the malignant characteristics of tumors. Previous transcriptomic analyses on UTUC primarily focused on bulk tissue samples, obscuring critical alterations in the transcriptomic spectrum within susceptible cell subpopulations. The advent of single-cell RNA sequencing (scRNA-seq) technology offers a remarkable opportunity to investigate the distinct characteristics of individual cells at a single-cell resolution (9). This technology has been employed to explore cell developmental trajectories, thus unraveling heterogeneities within the TME, and elucidating complex cellular classifications (10, 11). Recent advancements in single-cell analyses have allowed researchers to understand the specific cellular identities in UC. For instance, Fong and colleagues used scRNA-seq and T cell receptor (TCR) sequencing to uncover distinct cytotoxic CD4^+^ T cell subtypes within the BLCA TME, highlighting the pivotal role of the unique gene signature of cytotoxic CD4^+^ T cell in predicting the effectiveness of immunotherapy (12). Similarly, Shao et al. clarified intra-tumoral heterogeneity and delineated the pro-tumoral and immunosuppressive local microenvironment in UC using scRNA-seq, further supporting the development of therapeutic strategies (13). More recently, Li and their research team conducted scRNA-seq on UTUC tissues, and explored the tumor ecosystem of this disease. Their work elucidated differences in the TME between UTUC and BLCA and identified potential regulators of immune evasion in UTUC patients. However, there remains a significant gap in current research regarding the comprehensive mapping of single-cell transcriptomes in UTUC, and the transcriptomic distinctions between UTUC and normal ureteral tissues at the single-cell resolution have not been fully explored. Additionally, a systemic interpretation of the divergent cellular identities within the UTUC and BLCA TME represents an unmet research and clinical demand.

In this study, we conducted a comprehensive analysis using scRNA-seq analysis based on the 10X Genomics platform for three cases of UTUC and four normal ureteral tissues obtained from patients undergoing radical nephrectomy for kidney cancer without involvement of the collecting system. By integrating our in-house scRNA-seq data with publicly available datasets from previous BLCA studies, we aimed to address specific pivotal scientific inquiries. Firstly, we aimed to construct single-cell transcriptomic profiles for UTUC and normal ureteral tissues, delineating the transcriptional differences among distinct cell subsets within the microenvironment. Secondly, we sought to investigate the heterogeneity of transcriptional landscapes in identical cell types between UTUC and BLCA at the single-cell level, exploring both commonalities and distinctions. Lastly, this study aimed to identify potential biomarkers which can be promising clinical diagnostic markers and drug targets for UTUC. Our results provide deeper insights into the intricate molecular mechanisms underlying UTUC development and its relation to BLCA, contributing to the advancement of precision medicine in UTUC.

## Materials and methods

### Human specimens

Human ureteral tumor tissues were obtained from three UTUC cases undergoing surgical resection at Guangxi Medical University Cancer Hospital and were histopathologically confirmed by two independent pathologists. Normal ureteral samples were collected from four patients undergoing radical nephrectomy for kidney cancer at the same hospital, with the normal upper ureteral tissue harvested away from the tumor site. All sample collections were ethically approved by the ethics committee of Guangxi Medical University Cancer Hospital, and written informed consent was obtained from all patients. This study was approved by the Ethics Committee of Guangxi Medical University Cancer Hospital.

### Tissue processing

After excision, specimens were stored in a sterile centrifuge tube containing 1% RPMI 1640 (350–006-CL; WISENT) supplemented with antibiotics. The samples were transported to the laboratory for digestion within 30 minutes. Subsequently, we cleaned and trimmed the obtained tissue using 4°C Dulbecco’s phosphate-buffered saline (DPBS; 311–425-CL; WISENT). After the tissues were finely chopped, they were transferred to centrifuge tubes and centrifuged at 300g for 5 minutes at 4°C. The supernatant was discarded, and 10 ml of collagenase type IV (1.0 mg/ml; 17104019; Gibco) with DNaseI (0.2 mg/ml; 10104159001; Roche) was added to the samples. After 30 minutes of digestion in a 37°C water bath with manual agitation, 10 ml of DPBS solution was added to terminate the digestion. Subsequently, the cell supernatants were filtered through 100 μm nylon cell strainers. Red blood cells in the cell supernatants were removed using 5 ml of Red Blood Cell (RBC) Lysis Buffer (B250015; BioLegend) was added to the supernatants at a final concentration of 1x to remove red blood cells. Finally, the remaining cell supernatants were filtered with 40 μm nylon cell strainers, resulting in the single-cell suspensions of each sample. The quality of single-cell suspensions was assessed using trypan blue staining (0.4%; 420301; Gibco), with cell viability greater than 80% and no obvious cell clumps suitable for subsequent experiments.

### scRNA-seq details

Using the 10X Chromium 3’ Single Cell Platform, we performed scRNA-seq following the manufacturer’s protocol. Over 20,000 cells per lane were loaded onto a chip and partitioned into individual gel beads (GEMs) within the Chromium instrument. This was followed by cell lysis, barcoded reverse transcription of RNA within the droplets, emulsion breaking, amplification, fragmentation, and adapter and sample index addition. Pooled libraries were sequenced on the NovaSeq 6000 (PE150) platform. Finally, cDNA and library quality were assessed using the Qubit2.0 Fluorometer (14).

### Acquisition of online datasets

A total of four independent datasets were included in this study. Two BLCA scRNA-seq datasets (n=11, tumor = 7, para-carcinoma = 4) were obtained from the National Center for Biotechnology Information (NCBI) Gene Expression Omnibus (GEO) database (https://www.ncbi.nlm.nih.gov/geo/) under accession numbers GSE135337, and GSE129845. Additionally, bulk RNA-seq data for UTUC (n=20, tumor = 10, para-carcinoma = 10) was downloaded from the GEO database (accession number GSE47702), and bulk RNA-seq data for BLCA (n=429, tumor=409, para-carcinoma=19) was retrieved from The Cancer Genome Atlas (TCGA; https://portal.gdc.cancer.gov/) database.

### Analysis of scRNA-seq data

Fastq files were processed using Cell Ranger software (version 6.1.2, 10X Genomics) with default parameters and mapped to the human transcriptome GRCh38–2020 (https://support.10xgenomics.com/single-cell-gene-expression/software/downloads/latest). Seurat (version 4.2.0) was used to process single-cell data and perform subsequent analysis. For UTUC and normal ureteral samples, low-quality cells with less than 200 or more than 5000 expressed genes, and cells with more than 25% mitochondrial RNA content, were filtered out (15). For BLCA samples from online datasets, low-quality cells with fewer than 400 or more than 5000 expressed genes, as well as cells with more than 10% mitochondrial RNA content, were removed (16). SCTransform, RunPCA, and RunUMAP functions were applied for data normalization and dimensionality reduction, respectively (17). To correct for batch effects between different arrays, the harmony (version 0.1.1) package was employed (18). scHCL (version 0.1.1) and SingleR (version 1.10.0) packages aided in the identification of cell subpopulations, and cluster-specific marker genes were identified using the FindAllMarkers function of the Seurat package. Spearman correlations between different cell clusters were calculated using the Cor function of the R package stats (version 4.2.1) and visualized using the pheatmap package (version 1.0.12). To identify malignant cells within ureter and bladder epithelial cell populations, CopyKAT software version 0.1.0 was utilized to analyze the copy number variation (CNV) of each individual cell (19).

### Identifications of differentially expressed genes

Differential gene expression analysis in single-cell datasets was performed using the FindMarkers function in the Seurat package with a P-value<0.05 and |log_2_ expression Fold Change (log_2_FC)| >0.25 as cut-off criteria. DESeq2 (version 1.36.0), limma (version 3.52.4), and edgeR (version 3.38.4) packages were employed to identify DEGs in the TCGA BLCA cohort, with a P-value< 0.05 and |log_2_FC|>1 as thresholds. DEGs in the bulk RNA-seq data of the UTUC dataset from the GEO database were selected based on |log_2_FC| >1 and P-value < 0.05. The UpSetR (version 1.4.0) package was employed to visualize DEGs among different datasets.

### Gene ontology analysis, Kyoto encyclopedia of genes and genomes, and gene set variation analysis

GO function enrichment analysis and KEGG pathway enrichment analysis of the target genes in RNA-seq data were performed using the R package clusterProfiler (version 4.4.4), with results filtered using a P-value<0.05. Differences in biological processes and enrichment pathways between UTUC and BLCA tumor cells were explored through GSVA (version 1.44.5).

### Trajectory analysis

Single-cell pseudotime trajectories were constructed using monocle3 (version 0.2.3.0) and visualized in a two-dimensional scatter plot with UMAP1 and UMAP2. Pseudotime, indicating the sample’s developmental trajectory, was employed. Additionally, RNA velocity analysis and functional analysis of specific cell subpopulations were conducted to determine their developmental origins. RNA velocity analysis was performed using the Velocyto program (version 0.17.17) (20) and scVelo (version 0.2.4) (21). Files generated by Cell Ranger were converted into cellsorted.bam files using the Velocyto pipeline. Subsequently, UTUC and BLCA cells were isolated from the single-cell datasets and RNA velocities were predicted using the scVelo python program.

### Cell-cell interaction network analysis

Intercellular interaction analysis was performed using CellChat (version 1.5.0) (22). This analysis allowed us to identify the potential ligand-receptor interactions based on the expression patterns of ligands in one cell subtype and their corresponding receptors in other cell subtypes. The compareInteractions function was employed to assess the strength of interaction functionalities among samples. Visualization of cellular interactions was achieved through the netVisual_heatmap and netAnalysis_signalingRole_heatmap functions.

### Screening for potential therapeutic approaches for UTUC and BLCA

To assess the drug sensitivity of tumor cells in both BLCA and UTUC, we initially evaluated the expression levels of currently recognized drug targets for immunotherapy and molecularly targeted therapies. Subsequently, we used the R package Beyondcell (version 2.1.0) to identify drug sensibilities for the scRNA-seq data. We utilized the drug perturbation signature collection (PSc) database for this analysis, while also taking into account the correction of gene numbers detected per cell as recommended (23).

### Immunohistochemistry staining

Normal and cancer tissues were fixed in 4% paraformaldehyde for 48 hours and then embedded in paraffin. Subsequently, the paraffin-embedded tissues were sectioned into 4-μm-thick slices and dewaxed to water. For antigen retrieval, the sections were incubated in EDTA solution using medium heat for 8 minutes (to reach boiling point), followed by another 7 minutes at medium-low heat. To block endogenous peroxidase activity, the tissue sections were treated with 3% hydrogen peroxide and incubated in the dark at room temperature for 25 minutes. Followingly, tissue sections were evenly coated with a solution of 3% BSA, followed by a 30-minute blocking step at room temperature. After removing the blocking solution, the primary antibody, anti-MARCKS antibody (Abcam, ab52616, 1:200), was applied dropwise onto the sections, and were incubated at 4°C overnight. The corresponding horseradish peroxidase (HRP)-labeled secondary antibody (Servicebio, GB23303, 1:200) was incubated for 50 minutes at room temperature and further visualized using diaminobenzidine (DAB).

### Statistical analysis

All statistical analyses were performed using the R language(version 4.2.1). UMAP analysis was performed using the umap function from the uwot package (version 0.1.16). Statistical significance was set at P<0.05. Other detailed statistical tools, methods, and thresholds are described in the Methods section.

## Results

### Single-cell RNA sequencing reveals distinct cellular identities in UTUC and BLCA

**Figure 1:**
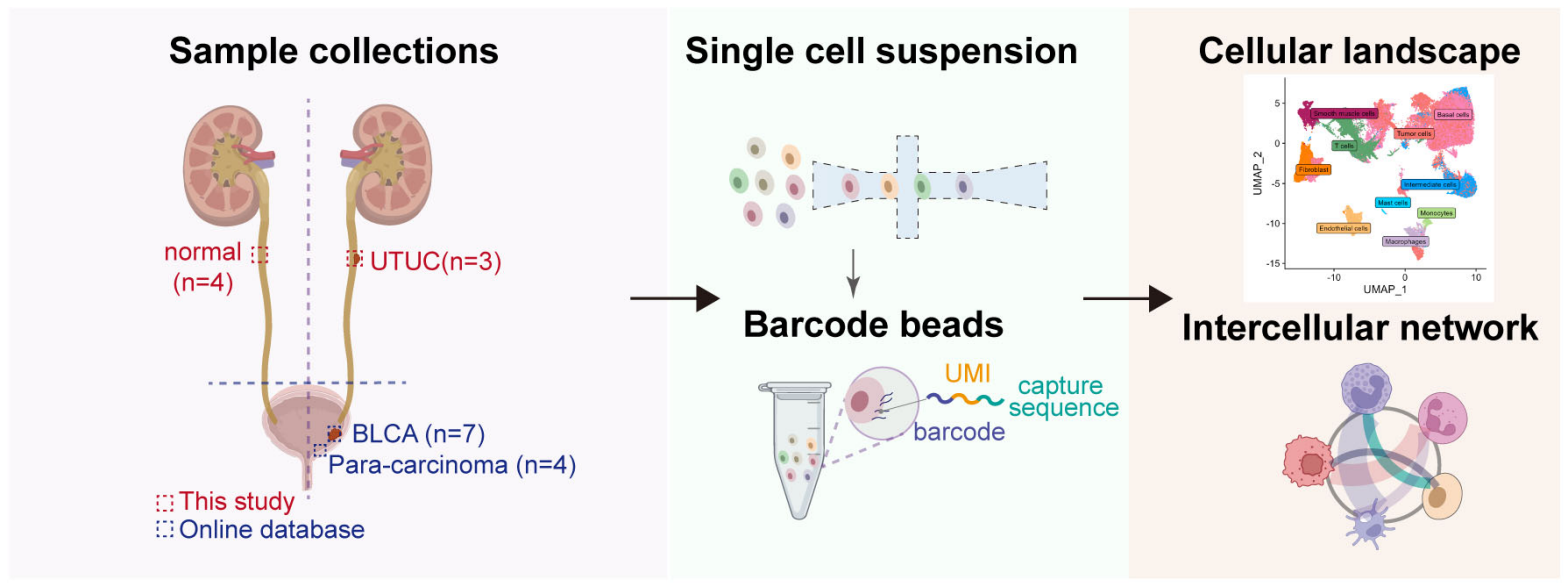
Workflow of the sample preparation process, sequencing and bioinformatic analysis of in-house UTUC and retrieval of publicly available BLCA scRNA-seq datasets.

**Figure 2:**
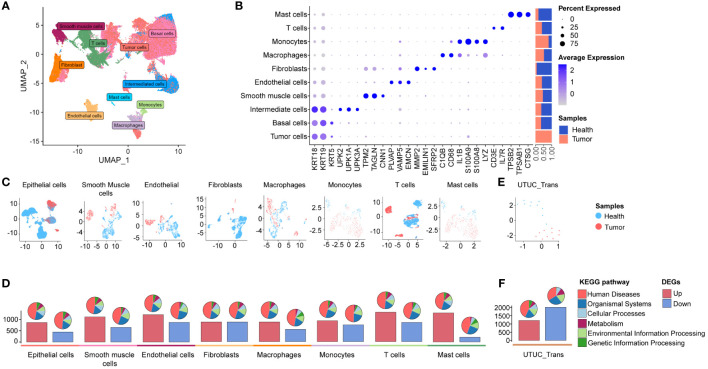
scRNA-seq and bulk RNA-seq profiling of UTUC. **(A)** UMAP displaying ten major cell clusters identified in the in-house UTUC scRNA-seq dataset. **(B)** Marker genes and relative proportions of sample origins for the ten major cell clusters. **(C)** Relative distribution of the major cell clusters. **(D)** Barplots showing the counts of DEGs between the cancer and health samples of each cell cluster in the UTUC scRNA-seq dataset. The pie plots at the top of the bar show the KEGG pathway enriched enrichment for each group of DEGs. **(E)** Relative distribution of GEO UTUC cohort. **(F)** Barplots showing the counts of DEGs between the UTUC and healthy cases of GEO UTUC cohort. The pie plots at the top of the bar show the KEGG pathway enrichment for each group of DEGs.

Our study commenced with the characterizations of the single-cell landscape of UTUC. As described previously, we collected seven surgical tissue samples, comprising three primary UTUC samples and four normal ureteral samples, for scRNA-seq analysis (**Figure 1**). Additionally, a cohort of bulk RNA-seq data for UTUC was obtained from the GEO database, which included cancer and normal tissues from ten UTUC patients (24). Following stringent quality control and batch effect removal, scRNA-seq analysis of UTUC yielded a total of 66,881 cells (**Supplementary Figure 1A**). Unsupervised clustering revealed the presence of ten major cell types (**Figure 2A**), including mast cells, T cells, monocytes, macrophages, fibroblasts, endothelial cells, smooth muscle cells, intermediate cells, basal cells, and tumor cells. Each cell type was characterized by canonical biomarkers, and the relative proportions of these cell types are shown in **Figure 2B**. Notably, CopyKAT was used to infer CNV, enabling the identification of aneuploid tumor cells (19). Cells with CNV alterations were classified as tumor cells and distinguished from normal epithelial cells in the scRNA-seq, specifically basal cells and intermediate cells. It is worth mentioning that epithelial cells, (both tumor and normal) and fibroblasts were the predominant cell types, with fibroblasts primarily originating from normal tissues. The distributions of each cell type and their respective sample origins can be visualized in the UMAP plot (**Figure 2C**).

Subsequently, we conducted an analysis of DEGs between cancer and normal tissues within the ten major cell types, based on their transcription profiles. Bar plots (**Figure 2D**, lower panel) depicted the total number of upregulated and downregulated DEGs, while pie plots categorize these DEGs into KEGG pathways (**Figure 2D**, upper panel), most of which are associated with “human disease”. Interestingly, the highest number of upregulated DEGs was observed in T cells from cancer compared to normal tissues, followed by upregulated DEGs in mast cells and endothelial cells. In contrast, downregulated DEGs in fibroblasts and endothelial cells exhibited a higher count compared to other cell types (**Figure 2D**, lower panel). These findings highlight substantial transcriptional reprogramming in various cellular identities within the UTUC tumor microenvironment, reshaping the local microenvironment of UTUC. Furthermore, we retrieved RNA-seq data for UTUC from the GEO database with accession number GSE47702 (**Figure 2E**), which revealed a total of 1203 upregulated and 2004 downregulated DEGs in UTUC tissues (**Figure 2F**).

**Figure 3:**
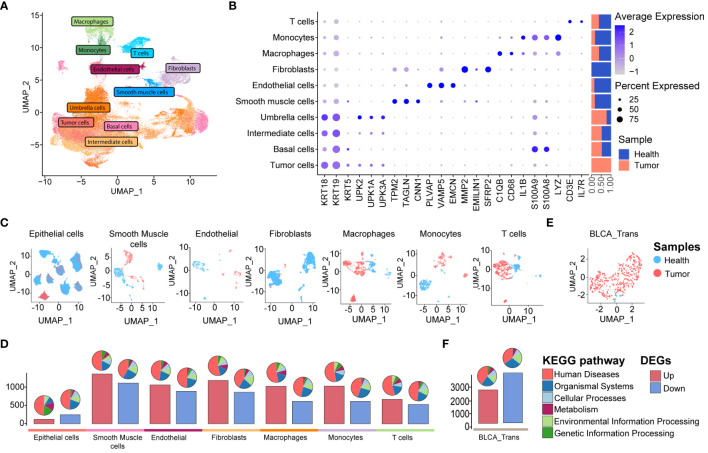
scRNA-seq and bulk RNA-seq profiling of BLCA. **(A)** UMAP displaying ten major cell clusters identified in the publicly available BLCA scRNA-seq dataset. **(B)** Marker genes and relative proportions of sample origins for the ten major cell clusters. **(C)** Relative distribution of the major cell clusters. **(D)** Barplots showing the counts of DEGs between the cancer and health samples of each cell cluster in the BLCA scRNA-seq dataset. The pie plots at the top of the bar show the KEGG pathway enrichment for each group of DEGs. **(E)** Relative distribution of TCGA BLCA cohort. **(F)** Barplots showing the counts of DEGs between the BLCA and healthy cases of TCGA BLCA cohort. The pie plots at the top of the bar show the KEGG pathway enrichment for each group of DEGs.

Next, we aimed to explore the transcriptomic commonalities and differences between UTUC and BLCA at the single-cell level, thereby investigating inter-tumor comparisons between these two diseases. To this end, we obtained four BLCA scRNA-seq datasets from the GEO database, including seven cancer and four para-tumor samples. The BLCA scRNA-seq data was processed following the procedures described above. After quality control and batch correction (details in **Supplementary Figure 1B**), 106,967 high-quality cells were retained for further analysis (**Figure 3A**). These cells were robustly categorized into ten major cell types through unsupervised clustering, encompassing almost identical cell types as observed in the UTUC scRNA-seq datasets, except for mast cells and the presence of basal cells. Cell type-specific marker genes and their relative proportions are presented in **Figure 3B**, with fibroblasts primarily originating from normal tissues, sharing similar characteristics with the UTUC scRNA dataset. Applying UMAP for dimensionality reduction (**Figure 3C**) clearly separated cells from normal and tumor tissues. DEG analysis revealed the highest number of DEGs between tumor and normal smooth muscle cells, followed by fibroblasts and macrophages (**Figure 3D**, lower panel). The associated KEGG pathway categories are visualized in the pie charts (**Figure 3D**, upper panel). Furthermore, we retrieved RNA-seq data for BLCA from the TCGA database (**Figure 3E**), which revealed a total of 2456 upregulated and 3705 downregulated DEGs in BLCA tissues relative to healthy tissues, with barplots showing the counts of DEGs and the pie plots at the top of the bar showing the KEGG pathway enrichment for each group of DEGs (**Figure 3F**).

Collectively, it reveals distinct cellular identities in these two cancer types. Despite superficial similarities, these cellular identities exhibited significant transcriptional differences, providing insight into the complex phenotypes of these cancers. This approach offers an unbiased portrayal of cellular heterogeneity and rationalizes the complex phenotypes of the two cancer types.

### BLCA and UTUC manifested completely different acquired immunological landscapes with varied cellular functional phenotypes of CD8^+^ T cells and Tregs

**Figure 4:**
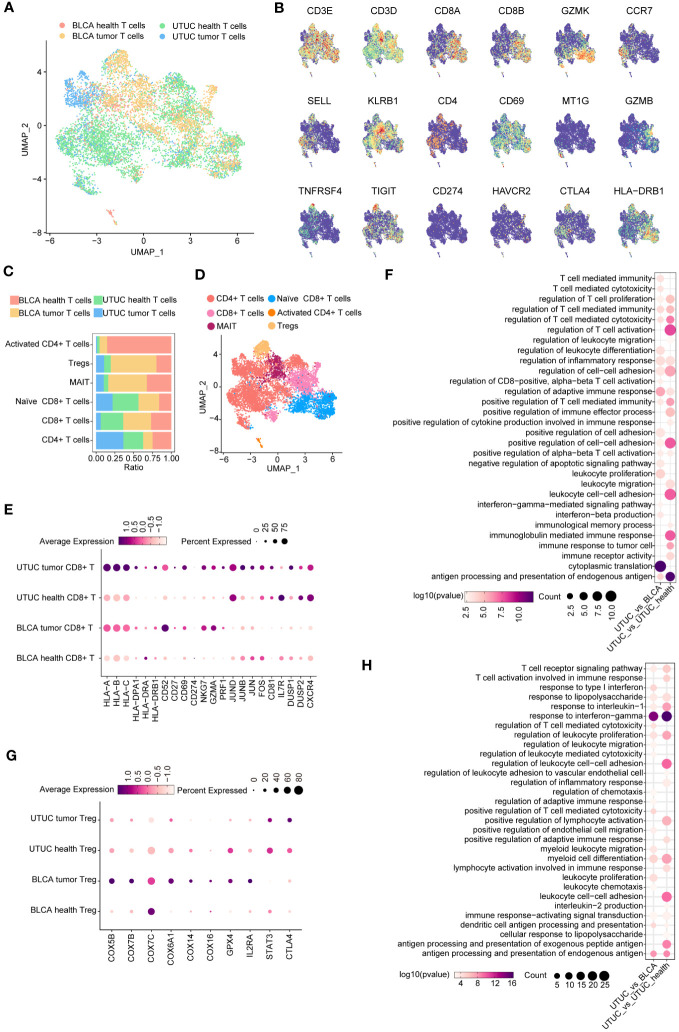
Identification and comparison of T cell subpopulations between the UTUC and BLCA. **(A)** UMAP showing the sample origins of T cell subpopulations. **(B)** The relative expression of marker genes in the T cell subpopulations. **(C)** Relative constituent ratio of each T cell subpopulation. **(D)** Relative distribution of six T cell subpopulations. **(E)** Bubble plot indicating the specific DEGs of CD8^+^ T cells in the UTUC relative to BLCA and normal ureter. **(F)** GO enrichment analysis of the upregulated DEGs of CD8^+^ T cells in UTUC relative to BLCA and normal ureter. **(G)** Bubble plot indicating the specific DEGs of Tregs in the UTUC and BLCA. **(H)** GO enrichment analysis of the downregulated DEGs of Tregs in UTUC relative to BLCA and normal ureter.

Tumor-infiltrating T lymphocytes (TILs) represent a heterogeneous cell subpopulation within the TME (12, 25). They exhibit significant plasticity and are associated with patient survival and therapeutic response in UC, making them a critical focus for distinguishing UTUC and BLCA. T cell subpopulations were identified and merged from scRNA datasets for UTUC and BLCA using canonical T cell markers CD3D and CD3E. UMAP analysis illustrated the sample origins (**Figure 4A**), revealing six distinct T cell subsets after dimensional reduction clustering; naive CD8^+^ T cells expressing GZMK (12), mucosal-associated invariant T (MAIT) cells expressing KLRB1 (26), regulatory T cells (Tregs) expressing well-known immune checkpoints (e.g., IL2RA, CTLA4, TIGIT, and TNFRSF4/9/18) (27, 28), activated CD4^+^ T cells expressing CD69 (12), canonical CD4^+^ T cells, and CD8^+^ T cells. Relative expression of selected marker genes and the constituent ratio of each T cell subset are shown in **Figures 4B**, **C**, respectively. The relative distribution of these six T cell subsets is shown in **Figure 4D**.

Intriguingly, we discovered a decreased presence of CD8^+^ T cells in UTUC compared to BLCA, with a lower proportion of these cells in UTUC cancer tissues. DEG analysis in CD8^+^ T cells revealed 72 upregulated and 148 downregulated genes between UTUC and normal ureter tissues. Notably, human leukocyte antigen (HLA) class I genes (HLA-A, HLA-B, HLA -C) and HLA class II genes (HLA-DPA1, HLA-DRA, HLA-DRB1) were significantly upregulated in UTUC CD8^+^ T cells compared to those in normal ureter tissues. Immune regulatory molecules involved in T cell activation, such as CD52 (29), CD27 (30), CD69 (31), and CD274 (32) were also upregulated (**Figure 4E**), suggesting enhanced antitumor immune responses mediated by CD8^+^ T cells (33). Furthermore, cytotoxic effector molecules NKG7, GZMA, and PRF1 were significantly elevated in these cell subsets (15) (**Figure 4E**). DEGs between CD8^+^ T cells from UTUC and BLCA tissues showed similar patterns, with upregulated expression of key genes involved in T cell activation, such as JUND, JUNB, JUN, and FOS (34), as well as other factors associated with CD8^+^ T cell effector functions CD81 (35), IL7R (36), DUSP1/2 (37), and CXCR4 (38) (**Figure 4E**). GO enrichment analyses confirmed the activation of immune‐related pathways, such as T cell-mediated cytotoxicity, regulation of adaptive immune response, and immune response to tumor cells (**Figure 4F**). These findings suggest that CD8^+^ T cells play a crucial role in the UTUC TME, contributing to the distinct disease phenotypes of UTUC and BLCA.

In contrast to UTUC, BLCA tumors exhibited a higher abundance of Tregs within their TME. To understand the impact of these Tregs on the local immune landscape, we investigated their transcriptomic differences between the two cancer types. DEG analysis revealed a significant disparity, with 131 genes upregulated and 179 downregulated in Tregs isolated from UTUC compared to BLCA. Notably, genes involved in mitochondrial oxidative phosphorylation showed downregulation in UTUC compared to BLCA (e.g., COX5B, COX7B, COX7C, COX6A1, COX14, and COX16), potentially affecting the energy metabolism and function of Tregs (39) (**Figure 4G**). Additionally, the decreased expression of GPX4 and IL2RA (40)implied specific functional states of the Tregs in UTUC (**Figure 4G**), suggesting an increased sensitivity to oxidative stress. Moreover, STAT3 and CTLA4 exhibited upregulated profiles in Tregs of UTUC (**Figure 4G**), which could hinder anti-tumor immunity (41), and suppress the production of immunosuppressive cytokines and metabolites, respectively (42). Enrichment analysis indicated that downregulated DEGs were predominantly associated with immune-suppressive effector functions (**Figure 4H**).

Myeloid cells are the predominant cell type in the TME and contribute significantly to the immunosuppressive microenvironment of UC (13). Therefore, we further characterized myeloid cell subtypes in the UTUC and BLCA TME, as displayed in **Supplementary Figure 2A**, which showed the sample origins. As such, three myeloid cell subtypes were identified: monocytes (CD68^+^/LYZ^+^/S100A8^+^/S100A9^+^), M1 macrophage (CCL5^+^/CCR7^+^/TNF^+^), and M2 macrophages (CD163^+^/MRC1^+^/MSR1^+^), with corresponding cell type markers shown in **Supplementary Figure 2B** and relative abundance in **Supplementary Figure 2C**. UMAP visualization for myeloid cell subtypes is presented in **Supplementary Figure 2D**. Notably, M1 macrophages were significantly more abundant in UTUC tumor tissues compared to normal ureter samples. DEG analysis identified 281 upregulated and 298 downregulated DEGs between UTUC and normal ureter-derived M1 macrophages. Upregulated DEGs included chemokines and receptors (CCL2, CCL3L1, CCL4L2, and CX3CR1) (**Supplementary Figure 2E**), known for their roles in recruiting myeloid-derived suppressor cells (MDSC) and prompting metastasis in certain cancer types (43). Additionally, inflammation-related genes (IL1RN, IL6, and IL32) (44, 45), and antigen presentation genes (CD14, HLA genes, and CD68) (46, 47) were upregulated. Notably, the expression of SPP1, associated with chemotherapy resistance in various cancer types (48) and poor response to cisplatin-based chemotherapy in muscle-invasive bladder cancer (MIBC) (49), was significantly elevated in UTUC tissues-derived M1 macrophages compared to normal ureter samples, shedding light on the underlying mechanism between UTUC chemotherapy resistance and M1 macrophages. DEGs between UTUC and BLCA-derived M1 macrophages identified 281 upregulated DEGs and 298 downregulated DEGs. Upregulated DEGs included chemokines (CXCL16, CCL2, CCL3, CCL4L2, and CCL4) and cytoskeleton-associated genes (ACTB, TUBB, DYNLL1, RACK1, and PFN1) (**Supplementary Figure 2E**) (50, 51), highlighting distinct immune modulatory and migratory capabilities between these two diseases.

In summary, we performed a comprehensive comparison of the immune microenvironments in UTUC and BLCA at the single-cell level, revealing differences in the relative abundance, composition, and functional status of immune cells. Notably, CD8^+^ T cells emerged as pivotal players in immunotherapy, displaying stronger anti-tumor activity in UTUC. Moreover, we observed significant variations in the functional states of Treg and M1 macrophages between the two diseases.

### Vein endothelial cells exert immunomodulatory functions in the UTUC TME

**Figure 5:**
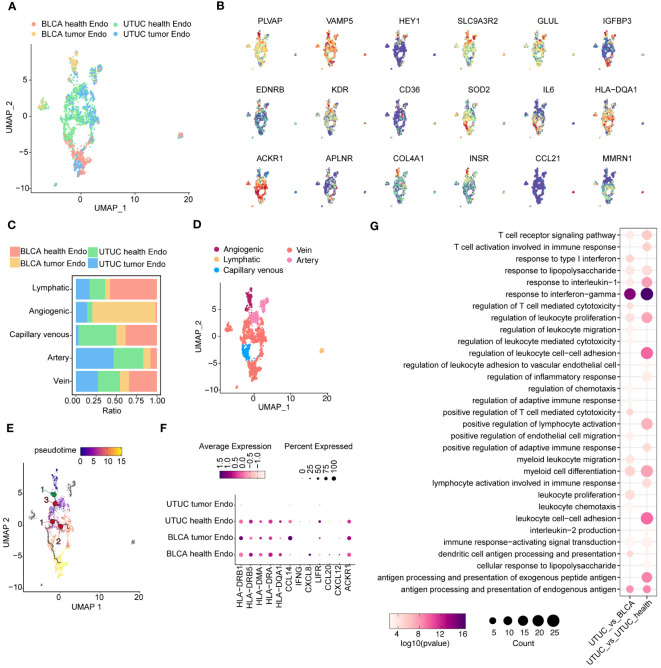
Identification and comparison of EC subpopulations in the UTUC and BLCA **(A)** UMAP showing the sample origins of EC subpopulations. **(B)** The relative expression of marker genes for EC subpopulations. **(C)** Relative constituent ratio of each EC subpopulation. **(D)** Relative distribution of five EC subpopulations. **(E)** Pseudotime trajectory for EC subpopulations. **(F)** Bubble plot indicating the specific DEGs of vein ECs in the UTUC compared to BLCA and normal ureter. **(G)** GO enrichment analysis for the downregulated vein ECs in UTUC compared to BLCA and normal ureter.

We obtained a total of 2446 high-quality vein ECs from both cancer and normal tissues (**Figure 5A**). Unsupervised clustering revealed five distinct subtypes, identified based on previously described cluster-specific marker genes (52, 53). These subtypes were vein ECs (ACKR1, IL6, HLA-DQA1), artery ECs (HEY1, IGFBP3, SLC9A3R2, GLUL), capillary venous ECs (CD36, EDNRB, KDR), angiogenic ECs (APLNR, COL4A1, INSR), and lymphatic ECs (CCL21, PROX1) (**Figure 5B**). Although all EC subtypes were present in the scRNA-seq datasets, their relative abundances varied (**Figure 5C**). A UMAP plot visually represented the relative distribution of EC subtypes (**Figure 5D**). Furthermore, we performed pseudotime analysis using Monocle 3 to determine the single-cell differentiation trajectory of different EC subtypes while validating their spatial distribution along the artery-capillary-venous tree (54). As expected, artery and vein EC subtypes were positioned at opposite ends of the trajectory, with capillary venous ECs enriched in the middle of the pseudotime trajectory (**Figure 5E**).

Growing evidence underscores the role of ECs in modulating immune homeostasis within the TME, particularly vein ECs. In comparison to vein ECs from normal ureter tissues, vein ECs in UTUC exhibited decreased expression levels of various immunomodulatory genes implicated in antigen presentation (HLA-DRB1, HLA-DRB5, HLA-DMA, HLA-DRA, HLA-DQA1) (55), immune cell recruitment (LIFR, CCL2, CXCL2, ACKR1) (56, 57) and anti-tumor inflammation (CCL14, IFNG) (58, 59) (**Figure 5F**). These findings suggest that vein ECs may contribute to the establishment of an immunosuppressive TME in UTUC. This observation is consistent with similar transcriptomic profiles observed in ECs from gastric cancer (53) and lung cancer (60), which may partially explain the potential synergistic efficacy of combining anti-angiogenic therapy with immune checkpoint inhibitors (ICIs) in the management of UTUC. Differential gene expression analysis of vein ECs between UTUC and BLCA revealed similar trends, with a decreased expression pattern of antigen presentation genes and chemokine-related genes in UTUC (**Figure 5F**). Subsequently, GO analysis of downregulated genes in UTUC-derived vein ECs confirmed impaired functions in the regulation of leukocyte cell-cell adhesion and response to interferon-gamma compared to vein ECs from normal ureter tissues (**Figure 5G**).

### Specific inflammatory fibroblasts phenotypes in the UTUC TME

**Figure 6:**
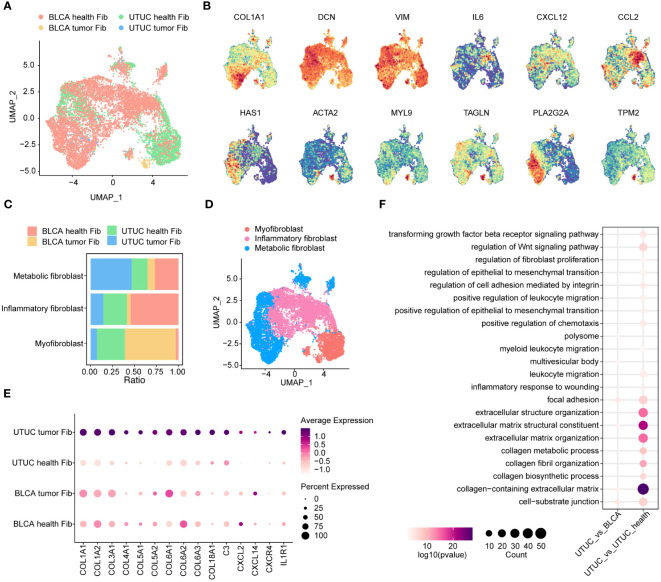
Identification and comparison of fibroblasts subpopulations in the UTUC and BLCA. **(A)** UMAP showing the sample origins of fibroblasts subpopulations. **(B)** The relative expression of marker genes for fibroblasts subpopulations. **(C)** Relative constituent ratio of each fibroblast subpopulation. **(D)** Relative distribution of three fibroblast subpopulations. **(E)** Bubble plot indicating the specific DEGs of inflammatory fibroblasts in the UTUC compared to BLCA and normal ureter. **(F)** GO enrichment analysis for the upregulated inflammatory fibroblasts in UTUC relative to BLCA and normal ureter.

Fibroblasts represent a phenotypically heterogeneous subset within the TME, with substantial differences across various cancer types, including BLCA (61). In our study, we focused on dissecting fibroblast subsets and extracting fibroblasts from both UTUC and BLCA datasets (**Figure 6A**). We identified a total of 9505 fibroblasts, which could be categorized into three subclusters: inflammatory fibroblasts, myofibroblasts, and metabolic fibroblasts. These subclusters exhibited augmented levels of COL1A1, DCN, and the mesenchymal marker VIM. Specifically, inflammatory fibroblasts showed exclusive upregulation of pro-inflammatory cytokines and chemokines, including IL-6, CCL2, and CXCL12 (14). Myo-fibroblasts were significantly enriched with smooth muscle-related genes such as ACTA2 and TAGLN, as well as genes encoding contractile proteins like MYL9, and TPM2 (62) (**Figure 6B**). Importantly, these subclusters were consistently present in both UTUC and BLCA patient cohorts (**Figures 6C, D**). Notably, we also identified, metabolic fibroblasts, previously described in scRNA-seq analysis of pancreatic ductal adenocarcinoma, characterized by elevated expression of marker gene PLA2G2A (63).

Inflammatory fibroblasts play an essential role as mediators of tumor-promoting inflammation, contributing to tumor growth, immune evasion, and metastatic dissemination through complex modulation of the immune profiles within the TME (64). In this context, we delved into investigating the transcriptomic differences of inflammatory fibroblasts between UTUC and normal ureter tissues. Our results revealed that collagen synthesis-related genes (e.g., COL1A1, COL1A2, COL3A1, COL4A1, COL5A1, COL5A2, COL6A1, COL6A2, COL6A3, and COL18A1) were upregulated in cancer tissues (**Figure 6E**). This suggests that inflammatory fibroblasts in UTUC possess advanced collagen production capabilities, which may contribute to cancer initiation, differentiation, and invasion. Furthermore, genes associated with cytokines, chemokines, and complement molecules are significantly enhanced in UTUC-derived inflammatory fibroblasts (**Figure 6E**). These molecules have been shown to collectively influence the local microenvironments by recruiting suppressive myeloid cells and Tregs (65). GO analysis of upregulated genes in UTUC-derived inflammatory fibroblasts revealed enhanced functions for collagen-containing ECM and the regulation of the Wnt signaling pathway (**Figure 6F**).

### Deciphering heterogeneity and differentiation trajectory of tumor cells in UTUC and BLCA

**Figure 7:**
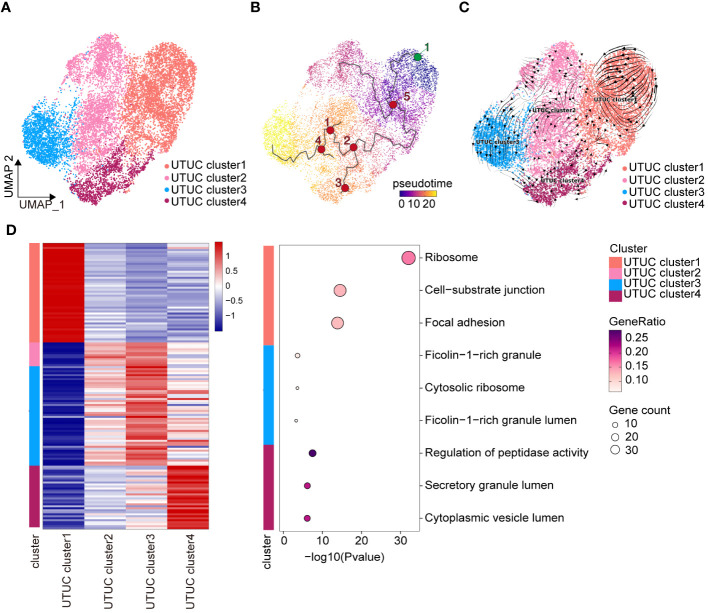
Heterogeneity and differentiation trajectory of tumor cells in UTUC. **(A)** UMAP displaying the four tumor cell subtypes in the UTUC. **(B)** Differentiation trajectory of the tumor cell lineages in the UTUC assessed by Monocle3. **(C)** Differentiation trajectory of the tumor cell lineages in the UTUC assessed by RNA velocity. **(D)** Four tumor cell subtypes in the UTUC and corresponding enriched pathways of their top 30 expressed gene.

Understanding the heterogeneity of inter- and intra-tumoral malignant cells is a crucial aspect of cancer research. Investigating the differences in the tumor cell populations between UTUC and BLCA is of significant clinical importance as it can lead to improvements in therapeutic responses and patient outcomes. We conducted an unsupervised clustering analysis to examine 12,696 tumor cells identified by the CopyKAT method. This analysis revealed the presence of four distinct tumor cell subtypes in UTUC (**Figure 7A**). To gain insights into the differentiation patterns and regulatory mechanisms of these four tumor cell populations in UTUC, we employed Monocle3 (**Figure 7B**) and the RNA velocity algorithm (**Figure 7C**). The results indicated that UTUC cluster 1 might serve as the starting point for the differentiation process. Notably, these four tumor cell subtypes in UTUC exhibited specific transcriptomic patterns, and we have presented the top 30 expressed genes and their corresponding enriched pathways in **Figure 7D**. Among these, the top 30 marker genes of UTUC cluster 1 were primarily associated with GO terms related to ribosomes, cell-substrate junctions, and focal adhesions, suggesting their high proliferative nature and active engagement in protein synthesis, along with strong interactions with the surrounding ECM. UTUC cluster 3, on the other hand, appeared to have immunoregulatory functions, as indicated by enriched terms such as ficolin-1-rich granule and lumen (66). Interestingly, marker genes of UTUC cluster 4 were associated with the regulation of peptidase activity, secretory granule lumen, and cytoplasmic vesicle lumen, suggesting that these tumor cells were characterized by active secretion and vesicular transport. Unfortunately, UTUC cluster 2 did not have sufficient marker genes involved in biological functions for further enrichment analysis. We identified five cell subtypes in the BLCA dataset (**Supplementary Figure 3A**). Employing Monocle3 (**Supplementary Figure 3B**) and RNA velocity (**Supplementary Figure 3C**), we reconstructed the developmental states of the tumor cell lineages in BLCA, which indicated that BLCA clusters 1 and 3 could potentially represent the starting points of their development trajectory. Subsequently, we conducted a similar analysis of the top 30 expressed genes associated with these five tumor cell subtypes in BLCA, and their GO terms are presented in **Supplementary Figure 3D**. This analysis highlighted the functionally heterogeneous nature of tumor cells in the BLCA TME.

To further the features of tumor cell subpopulations between BLCA and UTUC, correlation analysis was performed. Unexpectedly, extremely high similarities were observed between different tumor cell subpopulations of BLCA and UTUC, with a correlation coefficient between 0.65 and 1 (**Supplementary Figure 4A**). Among the, UTUC cluster 1 had the highest correlation with the BLCA clusters. Results of GSVA indicated that the tumor cell subpopulations of UTUC and BLCA could be perfectly distinguished (**Supplementary Figure 4B**), each with distinct biological characteristics and underlying molecular mechanisms.

### Construction of intercellular network of UTUC and BLCA ecosystems

**Figure 8:**
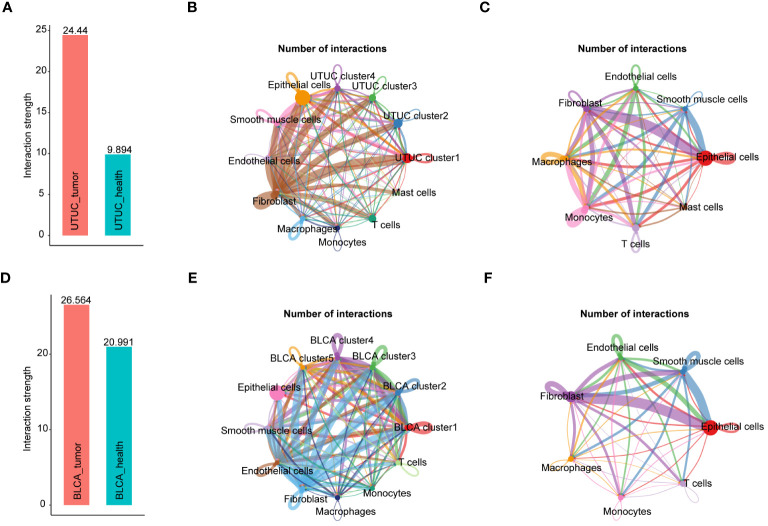
Intercellular network constructions of the UTUC and BLCA ecosystems. **(A)** Total interaction strengths in the UTUC ecosystems compared with corresponding normal tissue. **(B)** Circle plots showing the numbers of interactions in each cell subpopulation in UTUC. **(C)** Circle plots showing the numbers of interactions in each cell subpopulation in normal ureter tissues. **(D)** Total interaction strengths in the BLCA ecosystems compared with corresponding normal tissue **(E)** Circle plots showing the numbers of interactions in each cell subpopulation in BLCA. **(F)** Circle plots showing the numbers of interactions in each cell subpopulation in normal bladder tissues.

Next, CellChat package was used to analyze and visualize intercellular communications between UTUC and BLCA, in scRNA-seq datasets. It was found that cell subpopulations derived from UTUC had higher total interaction strengths compared with those from corresponding normal tissue (**Figures 8A**). Of particular interest, high interaction was observed between fibroblasts and other cell types in UTUC (**Figures 8B**) relative to normal ureter tissues (**Figures 8C**), especially for the tumor cells, demonstrating that fibroblasts may communicate with other cellular identities in the UTUC TME to regulate tumor growth, invasion, and metastasis. Besides, the number of interactions in BLCA was higher than in UTUC (**Figures 8D**). Interestingly, within BLCA samples, fibroblasts and ECs exhibited significantly more interactions with other cell types compared to normal tissues (**Figures 8E, F**). This suggests distinct communication patterns in the BLCA microenvironment. In UTUC, the intercellular network also varied, primarily driven by signaling pathways associated with ECM remodeling, involving collagen, thrombospondin (THBS), and fibronectin (FN1). In addition, signaling pathways associated with immune response and inflammation were also detected, e.g., CD99, CXCL, and ANGPTL (**Supplementary Figure 5A, B**), which had distinct interaction patterns compared to normal ureteral tissue (**Supplementary Figure 5C, D**). In BLCA, we observed robust interactions between ECs and tumor cells. Particularly, there was a strong interaction between ECs and tumor cells (**Supplementary Figure 5E**). This interaction pattern involved crucial signaling pathways such as growth differentiation factor (GDF), vascular endothelial growth factor (VEGF), platelet endothelial cell adhesion molecule 1 (PECAM1), and endothelial cell-selective adhesion molecule (ESAM) (**Supplementary Figure 5F**). The roles of these pathways in angiogenesis and inter-environmental communication have been extensively described in previous studies (67, 68), and these pathways displayed different signaling patterns relative to normal bladder tissues (**Supplementary Figure 5G, H**). In summary, the above results call for a deeper understanding of the role of fibroblasts in the UTUC, and the necessity of characterizing the interaction between fibroblasts and other cell types in the UTUC TME, which might improve our understanding differences in disease phenotypes between UTUC and BLCA.

Significantly, our observations revealed compromised immunomodulatory capabilities in ECs within the UTUC and BLCA TME compared to corresponding healthy controls, manifested by the downregulation of CCL and CXCL signaling pathways (**Supplementary Figure 5B, F**). This phenomenon unveils a noteworthy revelation: the receptor interactions between ECs and immune cells, present in normal tissues, are suppressed in UTUC and BLCA, resulting in the loss of immunomodulatory functions. Likewise, compelling evidence has documented the center stages of such two signaling pathways in the immunomodulation, chemotaxis and leukocyte trafficking, which are crucial for the maintenance of antitumor immune responses (69, 70).

### MARCKS is a prominent driver of UTUC progression and a critical factor driving phenotypic differences between UTUC and BLCA

**Figure 9:**
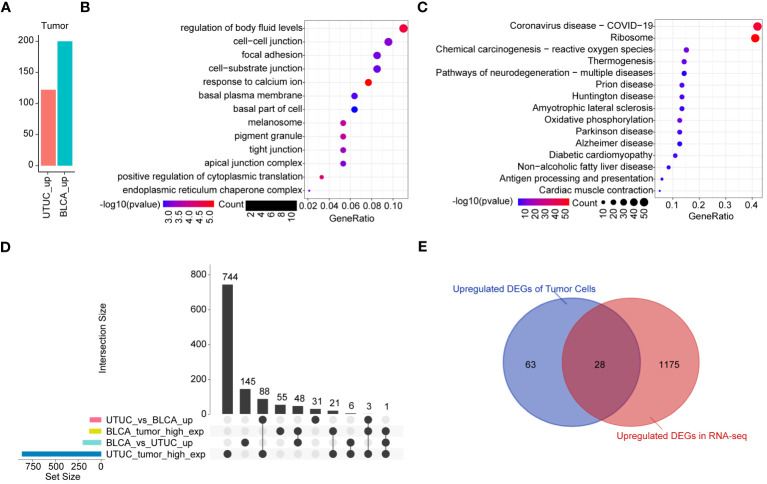
Identification of the key genes in the tumor cells of UTUC. **(A)** Identification of DEGs in tumor cells between BLCA and UTUC **(B)** KEGG pathway enrichment analysis of the upregulated DEGs in the tumor cells of UTUC. **(C)** KEGG pathway enrichment analysis of the upregulated DEGs in the tumor cells of BLCA. **(D)** Upset plot showing the intersection analysis between the groups compared **(E)** Venn plot showing upregulated DEGs in the tumor cells and RNA-seq data for UTUC.

Analysis of DEGs of tumor cells derived from BLCA and UTUC revealed 122 and 200 upregulated genes in the UTUC and BLCA, respectively (**Figure 9A**). KEGG pathway enrichment analysis showed that the DEGs upregulated in the UTUC were enriched in gap junction and tight junction, and their specific properties in terms of cell-to-cell interactions and adhesion were identified (**Figure 9B**). The involvement of leukocyte transendothelial migration and p53 signaling pathway confirmed that DEGs participated in immunomodulation and cell cycle regulation. Comparatively, the DEGs upregulated in BLCA were enriched in oxidative phosphorylation and ribosome signaling pathways (**Figure 9C**). To identify the key genes in the UTUC, intersection analysis between UTUC and BLCA scRNA-seq was carried out. A total of 88 DEGs were found to be upregulated in the tumor cells of UTUC relative to those in BLCA and normal ureter tissues. Among them, three DEGs were also upregulated in the tumor cells of BLCA (**Figure 9D**). Next, RNA-seq data of UTUC was obtained from GEO database with accession number of GSE47002. After identifying DEGs between UTUC and normal samples using a threshold of P-value< 0.05 and |log2FC|>1, an intersection analysis was conducted on the upregulated DEGs from both the scRNA-seq and RNA-seq datasets. This analysis identified 28 key genes that were closely linked to tumor cells in UTUC (**Figure 9E**).

**Figure 10:**
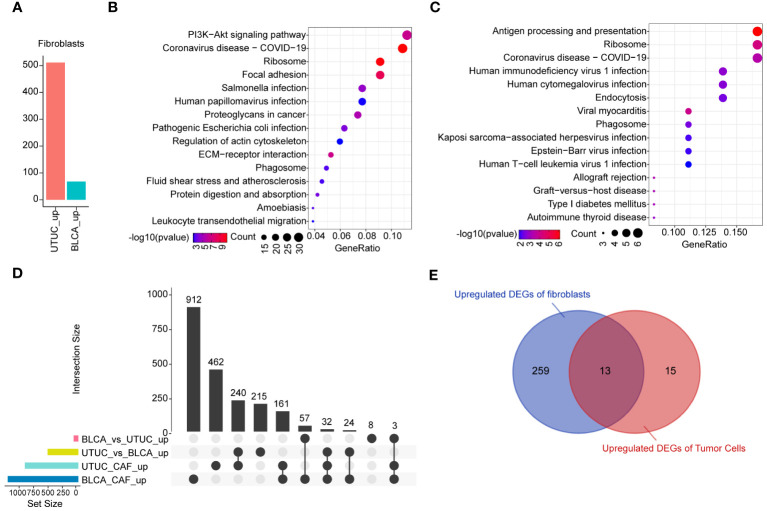
Identification of the key genes in the fibroblasts of UTUC. **(A)** Identification of DEGs in fibroblasts between BLCA and UTUC. **(B)** KEGG pathway enrichment analysis of the upregulated DEGs in the fibroblasts of UTUC. **(C)** KEGG pathway enrichment analysis of the upregulated DEGs in the fibroblasts of BLCA. **(D)** Upset plot showing the intersection analysis between the groups compared. **(E)** Venn plot showing upregulated DEGs in the tumor cells and fibroblasts for UTUC.

**Figure 11:**
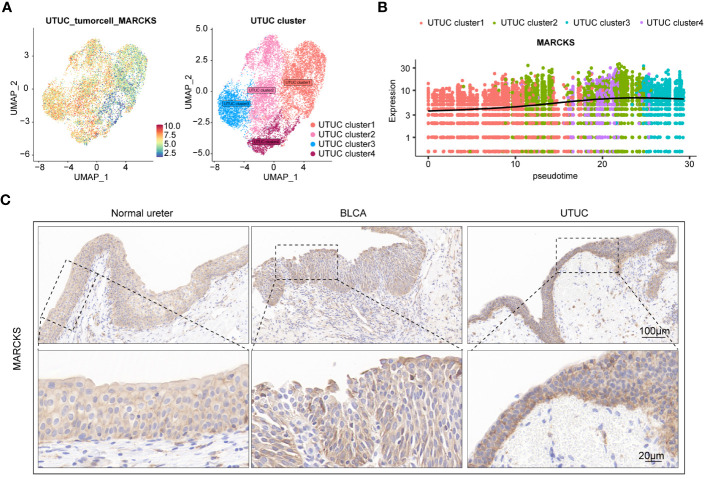
MARCKS is a prominent driver of UTUC progression. **(A)** Expression levels of MARCKS in tumor cell subpopulations of UTUC. **(B)** Expression levels of MARCKS during the differentiation of tumor cell subpopulations of UTUC. **(C)** IHC showing upregulated MARCKS in the UTUC tissues relative to BLCA and normal ureter.

As previously described, fibroblasts participate in the pathogenesis of UTUC pathogenesis. Analysis of the DEGs of fibroblasts derived from BLCA and UTUC identified 511 and 68 upregulated DEGs in the UTUC and BLCA, respectively (**Figure 10A**). KEGG pathway enrichment analysis indicated that the DEGs upregulated in the fibroblasts of UTUC were enriched in the PI3K-Akt signaling pathway, ECM-receptor interaction, and leukocyte transendothelial migration (**Figure 10B**), whereas the DEGs upregulated in the fibroblasts of BLCA were enriched in antigen processing and presentation, ribosome, and endocytosis (**Figure 10C**). Intersection analysis between fibroblasts of UTUC and BLCA scRNA-seq dataset identified 272 key genes that were strongly associated with fibroblasts in the UTUC (**Figure 10D**). Analysis of the intersection of the 28 key genes closely associated with tumor cells in the UTUC revealed 13 key genes (**Figure 10E**), including ATXN2L, RAN, GALNT1, IGFBP3, S100A11, SEPT9, MARCKS, HPGD, ACTG1, CLDN1, TUBA1C, UPK1B, and YPEL3, which showed differential high expression in tumor cells of UTUC (**Supplementary Figure 6**). Among them, MARCKS was ubiquitously overexpressed in all cancer-tissues derived cell subpopulations (**Figure 11A**), and its expression level gradually increased with the differentiation of UTUC tumor cells (**Figure 11B**). Results of IHC examination further confirmed the upregulation of MARCKS in UTUC tissues relative to BLCA and normal ureter (**Figure 11C**). Based on this, we postulated that MARCKS may be a key driver of UTUC progression, and an important factor mediating phenotypic differences between UTUC and BLCA.

### Predictions of antitumor drug efficacy and explorations for the potential therapeutic agents

As previously described, drugs commonly utilized in the management of BLCA show reduced efficacy in cases with UTUC. This underscores the urgent need for focused scientific endeavors to enhance our understanding and implementation of precision medicine in UTUC management. For this purpose, we visualized the expression levels of current immunotherapeutic and targeted therapeutic targets in UTUC and BLCA cells, and predicted the clinical therapeutic effects of such drugs (**Supplementary Figure 7A**). Our analysis revealed that most therapeutic target genes were expressed at low levels in UTUC tumor cells. However, ERBB2, ERBB3, and PARP1 showed relatively higher expression, suggesting that drugs targeting these genes could be potentially effective. This is supported by promising results from clinical trials and experimental data demonstrating the efficacy of such inhibitors in urothelial carcinoma treatment (71, 72).

Next, the R package Beyondcell we used to identify candidate drugs for BLCA and UTUC. Results indicated that Mevastatin, SKF-104078, and JNJ-10191584 exerted antitumor effects on BLCA (**Supplementary Figure 7B**). Mevastatin is a widely used medication and is commonly prescribed for patients with hypercholesterolemia and cardiovascular diseases. In recent years, its scope of application has extended beyond cholesterol regulation to include cancer and inflammatory conditions (73). Likewise, SKF-104078 is a compound classified as a non-classical monocarboxylate transporter (MCT) inhibitor, which might interfere with tumor cell growth and survival by regulating cellular metabolism, energy balance, and extracellular acid-base homeostasis (74). On the other hand, JNJ-10191584 is a small molecule compound categorized as the histone deacetylase (HDAC) inhibitor, which can potentially disrupt cellular epigenetic modifications to induce cell cycle arrest, apoptosis, and differentiation of tumor cells (75). Notably, mycophenolate mofetil, epigallocatechin, and epigallocatechin have been predicted to have anti-tumor effects in UTUC (**Supplementary Figure 7C**). Mycophenolate mofetil, FDA-approved for autoimmune diseases, is under investigation as an anticancer agent and has shown good central nervous system (CNS) penetrance in patients with conditions like neurosarcoidosis (76). Epigallocatechin is a naturally occurring catechin compound found in tea leaves, with potent antioxidant and anti-inflammatory properties. It has been extensively investigated for its potential health and medicinal benefits. Some studies have explored its potential anticancer effects, indicating that it can regulate the growth and spread of cancer cells (77). SCH-23390 is a selective dopamine D1 receptor antagonist, however, its role in the anti-tumor functions has never been appreciated previously.

## Discussion

To the best of our knowledge, this is the first study to present the comprehensive single-cell atlas of UTUC and normal ureter. Several studies have conducted scRNA-seq analyses on UTUC. However, the lack of normal tissue data and diverse cellular identities has limited our understanding of the specific features that distinguish UTUC from healthy individuals (15). To address this gap, we conducted this investigation to explore the molecular processes underlying UTUC development. Moreover, we compared UTUC and BLCA at the single-cell resolutions, the first study of this kind. Although both diseases belong to UC, UTUC is mainly characterized by a more aggressive and less differentiated phenotype relative to BLCA (78). This study, therefore, provides new perspectives and reveal the unique molecular signatures of UTUC and BLCA, presenting ideas for developing more effective therapeutic interventions for UTUC.

Herein, we have identified a characteristic immune landscape in UTUC that is distinct from both BLCA and normal ureter. Notably, CD8^+^ T cells in the UTUC were activated as evidenced by the enhanced anti-tumor immune responses and high expression of cytotoxic effector molecules. However, the relative abundance of such pivotal anti-tumor effector cells was decreased, indicating a characteristic feature of T cell exclusion or poor tumor infiltration or in UTUC, which have been documented as essential immune evasion mechanisms in melanoma (79). Moreover, the proportion of Tregs was increased in the UTUC microenvironment, accompanied by upregulated levels of the immunosuppressive molecule CTLA-4. Overexpressed CTLA-4 is known to disrupt co-stimulatory signaling in both antigen-presenting cells and effector cells, effectively suppressing immune responses. Taken together, these findings suggest a distinct immunological landscape in UTUC compared to BLCA, characterized by a relatively “cold” microenvironment. In addition, we found increased expression of SPP1 in UTUC-derived M1 macrophages relative to normal ureter, and SPP1 was recently reported to possess immunosuppressive properties and to be positively correlated with epithelial-mesenchymal transition (EMT) markers (80). Intriguingly, our analysis indicates that UTUC-derived M1 macrophages may possess enhanced chemotactic ability compared to their BLCA counterparts. Our findings shed light on the unique immunosuppressive landscape shaping UTUC and offer valuable insights into why UTUC patients respond differently to the same treatments compared to BLCA. This study reveals the distinct immune microenvironment of UTUC relative to BLCA and healthy ureters, highlighting key differences that can be leveraged to develop targeted therapies and personalized treatment strategies for UTUC.

Tumor cells actively recruit supportive stromal cells from nearby tissues, which influence tumor initiation and growth. This highlights the impact of stromal cells on the TME, which contains various components like ECs, fibroblasts, and adipocytes. Importantly, the TME exhibits significant heterogeneity across different tumor types (81). Recent advancements in single-cell multi-omics profiling and integrative bioinformatics have revealed the specific immunomodulatory roles of these stromal cells within both healthy organ (82) and the TME (83). Studies have shown that neoplastic transformation often coincides with the remodeling of parenchyma and alterations in the stroma regions, which lead to the reestablishment of TME, the latter of which has been demonstrated to promote tumor growth and affect the clinical outcomes of anti-tumor therapies (84).

In our study, the specific patterns identified were investigated by exploring the transcriptomic alterations of various EC subpopulations in the UTUC TME. Differentiation analysis followed the trajectory of the artery-capillary-venous tree, while the distributions of artery and vein ECs were located in the opposite ends of the trajectory. Of note, we infer that in UTUC, the ECs exhibited lower expression levels of several crucial immunomodulatory genes compared to both normal ureter and BLCA. These genes play intricate roles in processes like antigen presentation, immune cell recruitment, and anti-tumor inflammatory responses. Our analysis into the downregulated DEGs further revealed a significant enrichment in the immune-related pathways. Interestingly, this transcriptomic shift in UTUC vein ECs mirrors similar changes observed in breast (52) and lung cancers vein ECs (85). Additionally, a parallel observation was made for vein ECs in murine lymph nodes, which also demonstrated immune recruitment properties (85). Previous research suggests that the slower blood flow and reduced shear stress in veins might create an environment conducive to interactions between ECs and immune cells (52), possibly explaining the immunomodulatory role of vein ECs in the TME. The discovery of these alterations in vein ECs in this study not only deepens our understanding of UTUC but also suggests that antiangiogenic treatments may be effective in the managements of this disease (86). Similarly, we discovered that various subtypes of fibroblasts in the UTUC TME exhibited distinctive features. Inflammatory fibroblasts within the microenvironment expressed a distinct set of genes related to collagen synthesis and showed significant interactions, particularly with tumor cells. These interactions involved key intercellular signaling molecules like collagen, THBS, FN1, and PTN, all known contributors to ECM formation and remodeling (82, 83). This suggests that fibroblasts in the TME play a crucial role in coordinating ECM remodeling. They do so by releasing substantial amounts of collagen and fibronectin, thereby facilitating tumor invasion and migration. In addition, signaling patterns associated with immunoregulation and inflammation were also involved, suggesting that in the context of UTUC TME, fibroblasts can promote tumorigenesis and progression by creating a pro-inflammatory and immunosuppressive microenvironment (87, 88).

Compared to BLCA, UTUC is a relatively rare malignancy. It is distinguished by its unique genomic, biological, and clinical features. Unfortunately, no effective strategies currently exist for diagnosing and treating UTUC, with initial diagnosis typically relying on ureteroscopic biopsy (89). However, this procedure is associated with several complications, including bleeding, infection, ureteral injury, and bladder implantation. Existing treatment options for UTUC, such as surgical resection, are limited by some inherent risks such as renal dysfunction and surgical complications (90). With the rapid advancement of multi-omics technologies, integrative analysis has emerged as a powerful tool for exploring biomarkers in UTUC. Qu et al., using plasma proteomic analysis, observed that, in comparison to normal ureteral tissue and non-muscle-invasive UTUCs, HPCAL1 was overexpressed in muscle-invasive UTUC samples, while TST presented the opposite trend. A classifier based on these two differentially expressed proteins could potentially serve as a predictive model to distinguish such two types of UTUCs (91). Fujii and their team analyzed the pairwise relationships between mutations and copy number alterations in 188 non-hypermutated UTUC samples, revealing four distinct subtypes with unique co-alteration/mutually exclusive patterns characterized by the presence/absence of alterations in TP53 or MDM2, RAS, and FGFR3, further indicating that the detections of such common mutation sites using urinary sediment sequencing has potential value as both a prognostic biomarker and a diagnostic tool in UTUC (92).

In this study, we comprehensively screened key genes involved in both fibroblasts and tumor cells, identifying MARCKS proteins as potential diagnostic markers for UTUC. Moreover, MARCKS were overexpressed in all tumor tissue-derived cell subpopulations, with its expression levels progressively increasing with the differentiation of UTUC tumor cells. Moreover, MARCKS were found to be a crucial factor for distinguishing between UTUC and BLCA. The expression profile of MARCKS across clinical samples was explored through IHC. MARCKS, known as myristoylated alanine-rich C kinase substrate, is an autophagy-related gene. Previous investigations have identified it as a diagnostic marker for gastric cancer (93), and has also been shown to enhance cell migration and invasion by interacting with F-actin and ECM degradation (94). Similarly, Chen et al. reported that MARCKS is a potential target in kidney cancer, and its expression is positively correlated with tumor grade. Genetic and pharmacological suppression of MARCKS in high-grade renal cell carcinoma cell lines resulted in inhibited cell proliferation and migration (95). Similarly, our findings underscore the diagnostic potential of MARCKS in UTUC, positioning it as a promising therapeutic target for UTUC.

Based on our analysis, we hypothesize that MARCKS plays a pivotal role in promoting the interactions between fibroblasts and tumor cells in the UTUC TME. However, the dialogue mechanism between these two cellular identities and its exploration regarding malignant phenotypes require further investigation. Undoubtedly, at the single-cell resolution, we could observe transcriptional changes in cells at a higher dimension, which holds significant implications for the screening of tumor biomarkers.

## Conclusion

In conclusion, our study significantly advances the understanding of single-cell transcriptomics in UTUC and BLCA, providing valuable insights for diagnosis and treatment. The identified cell subtypes and immune landscapes offer potential diagnostic and therapeutic targets, particularly in the promising field of UTUC immunotherapy. To deepen our understanding of disease progression and therapeutic interventions, further research must explore dynamic alterations in tumor cell populations and interactions, both *in vivo* and *in vitro*, offering promising insights for personalized UTUC treatment strategies and improved patient outcomes.

## Data availability statement

The scRNA-seq data reported in this study have been deposited in NGDC OMIX database (OMIX ID: OMIX004911; https://ngdc.cncb.ac.cn/omix/preview/GSiidU01).

## Ethics statement

The studies involving humans were approved by Guangxi Medical University Cancer Hospital Ethical Review Committee. The studies were conducted in accordance with the local legislation and institutional requirements. The participants provided their written informed consent to participate in this study.

## Author contributions

QZ: Data curation, Formal Analysis, Funding acquisition, Validation, Writing – original draft. CW: Data curation, Formal Analysis, Validation, Visualization, Writing – original draft. MQ: Data curation, Formal Analysis, Writing – original draft. YY: Data curation, Writing – original draft. YM: Formal Analysis, Writing – original draft. QM: Data curation, Writing – original draft. GY: Data curation, Writing – original draft. GF: Data curation, Writing – original draft. RL: Data curation, Writing – original draft. SX: Data curation, Writing – original draft. JW: Data curation, Writing – original draft. SC: Conceptualization, Investigation, Supervision, Validation, Writing – review & editing. SW: Conceptualization, Investigation, Resources, Supervision, Writing – review & editing. ZM: Conceptualization, Funding acquisition, Investigation, Project administration, Resources, Supervision, Writing – review & editing.

## Supplementary material

Supplementary Figure 1 Quality control of scRNA-seq data. **(A)** Violin plots showing the distribution and the number of detected genes per cell, read counts per cell, and the proportion of mitochondrial genes per cell before and after quality control of scRNA-seq data for UTUC. **(B)** Violin plots showing the distribution for the number of detected genes per cell, read counts per cell, and the proportion of mitochondrial genes per cell before and after quality control of scRNA-seq data for BLCA.

Supplementary Figure 2 Identification and comparison of myeloid cell subtypes in the UTUC and BLCA. **(A)** UMAP showing the sample origins of myeloid cell subtypes. **(B)** The relative expression of marker genes for myeloid cell subtypes. **(C)** Relative constituent ratio of each myeloid cell subtypes. **(D)** Relative distribution of three myeloid cell subtypes. **(E)** Bubble plot indicating the specific DEGs of M1 macrophage in the UTUC relative to BLCA and normal ureter.

Supplementary Figure 3 Heterogeneity and differentiation trajectory of tumor cells in BLCA. **(A)** UMAP revealing four tumor cell subtypes in the BLCA. **(B)** Differentiation trajectory of the tumor cell lineages in the BLCA assessed by Monocle3. **(C)** Differentiation trajectory of the tumor cell lineages in the BLCA assessed by RNA velocity. **(D)** Four tumor cell subtypes in the BLCA and corresponding enriched pathways of their top 30 expressed gene.

Supplementary Figure 4 Similarities and disparities of the tumor cell subpopulations between BLCA and UTUC. **(A)** Correlation analysis of the transcriptomes of tumor cell subpopulations in BLCA and UTUC. **(B)** GSVA of the tumor cell subpopulations in UTUC and BLCA.

Supplementary Figure 5 Intercellular network construction based on the CellChat. **(A, B)** Intercellular network among various cell subtypes in UTUC and corresponding signaling pattern. **(C, D)** Intercellular network among various cell subtypes in normal ureter tissues and corresponding signaling pattern. (E-F) Intercellular network among various cell subtypes in BLCA and corresponding signaling pattern. **(G, H)** Intercellular network among various cell subtypes in normal bladder tissues and corresponding signaling pattern.

Supplementary Figure 6 Comparison of the relative expression pattern of 28 key DEGs closely associated with tumor cells between UTUC and normal ureter tissues.

Supplementary Figure 7 Predictions of antitumor drug efficacy and explorations for the potential therapeutic agents. **(A)** Expression levels of current immunotherapeutic and targeted therapeutic targets in tumor and normal epithelial cell of UTUC and BLCA. **(B)** Candidate drug prediction for BLCA using R package Beyondcell. **(C)** Candidate drug prediction for UTUC using R package Beyondcell.
