# Supplementary figures and images for: Investigating cellular similarities and differences between upper tract urothelial carcinoma and bladder urothelial carcinoma using single-cell sequencing
Source: Front Immunol. 2024 Jun 6;15:1298087. doi: 10.3389/fimmu.2024.1298087 (PMC11187293; doi:10.3389/fimmu.2024.1298087)

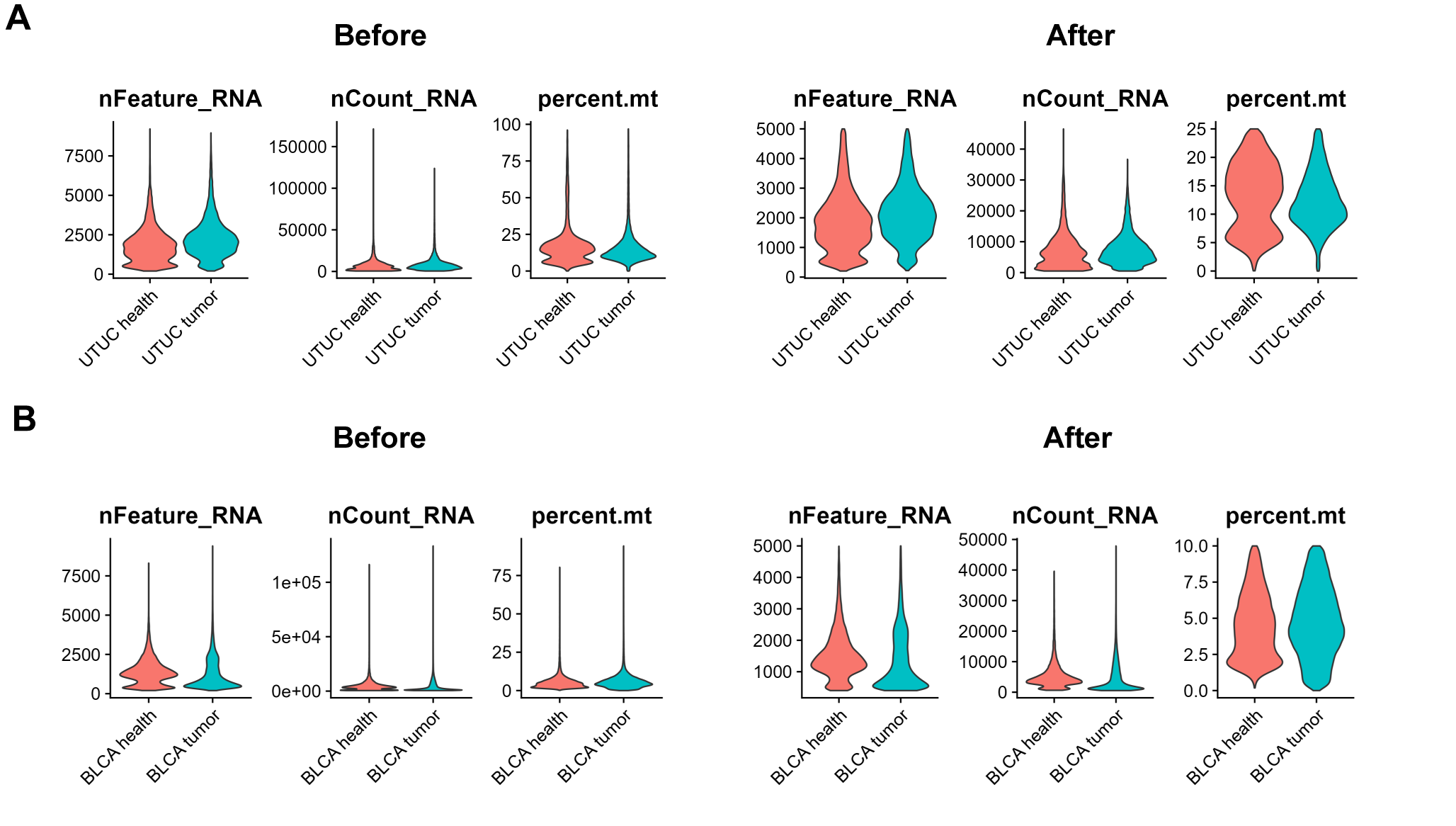

Supplement: Supplementary Figure 1 — Quality control of scRNA-seq data. (A) Violin plots showing the distribution and the number of detected genes per cell, read counts per cell, and the proportion of mitochondrial genes per cell before and after quality control of scRNA-seq data for UTUC. (B) Violin plots showing the distribution for the number of detected genes per cell, read counts per cell, and the proportion of mitochondrial genes per cell before and after quality control of scRNA-seq data for BLCA. [file Image_1.tif]

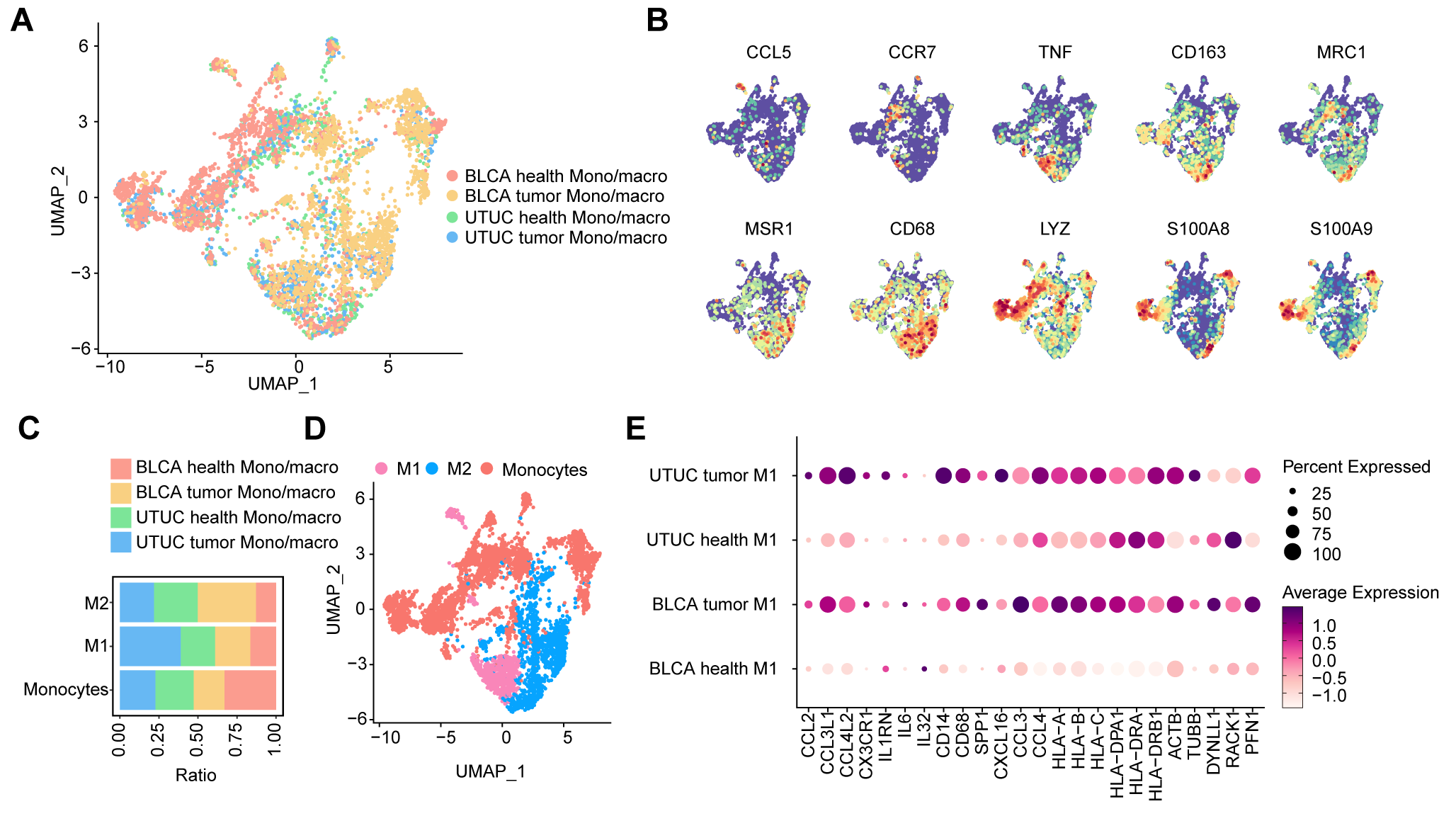

Supplement: Supplementary Figure 2 — Identification and comparison of myeloid cell subtypes in the UTUC and BLCA. (A) UMAP showing the sample origins of myeloid cell subtypes. (B) The relative expression of marker genes for myeloid cell subtypes. (C) Relative constituent ratio of each myeloid cell subtypes. (D) Relative distribution of three myeloid cell subtypes. (E) Bubble plot indicating the specific DEGs of M1 macrophage in the UTUC relative to BLCA and normal ureter. [file Image_2.tif]

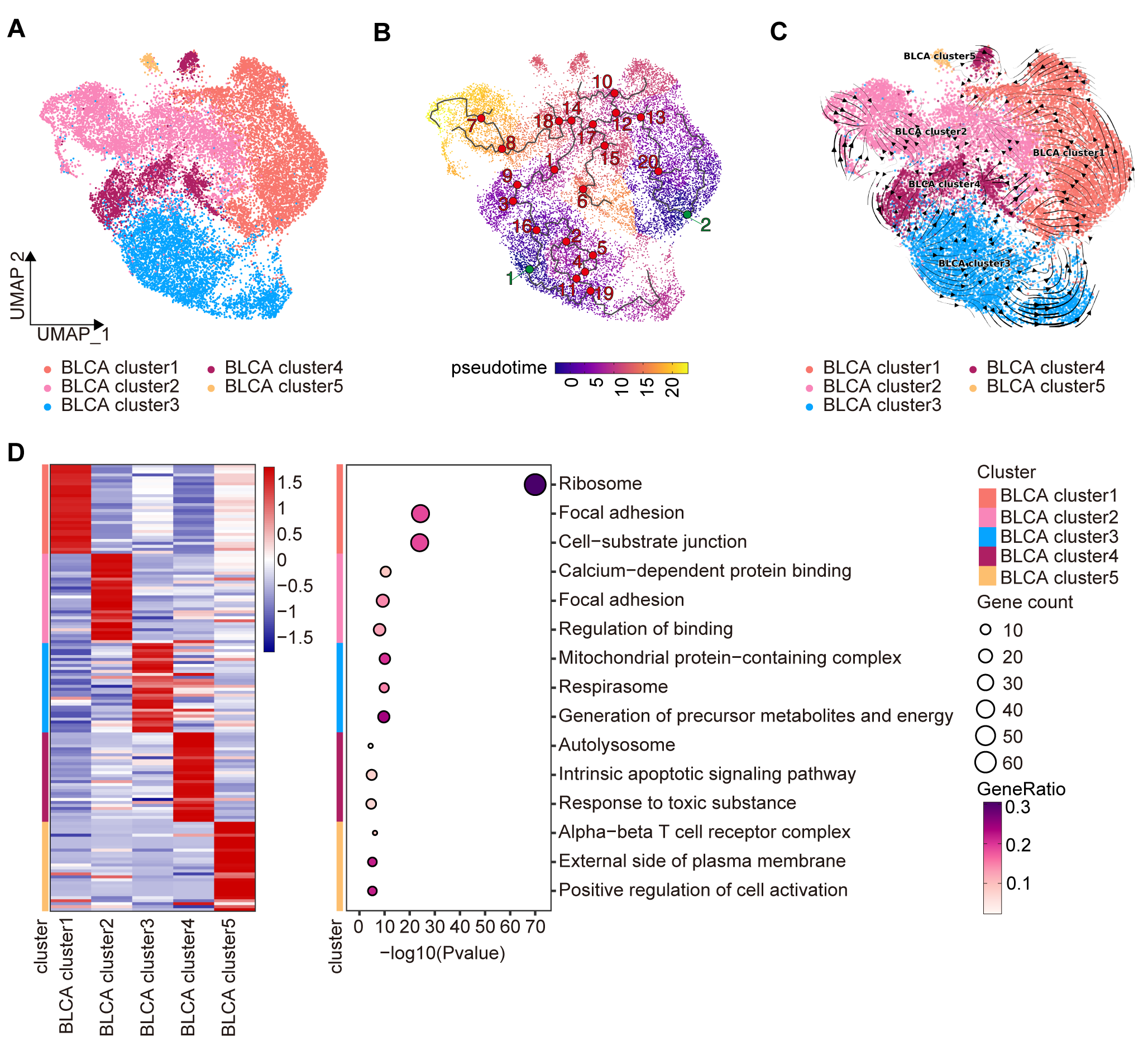

Supplement: Supplementary Figure 3 — Heterogeneity and differentiation trajectory of tumor cells in BLCA. (A) UMAP revealing four tumor cell subtypes in the BLCA. (B) Differentiation trajectory of the tumor cell lineages in the BLCA assessed by Monocle3. (C) Differentiation trajectory of the tumor cell lineages in the BLCA assessed by RNA velocity. (D) Four tumor cell subtypes in the BLCA and corresponding enriched pathways of their top 30 expressed gene. [file Image_3.tif]

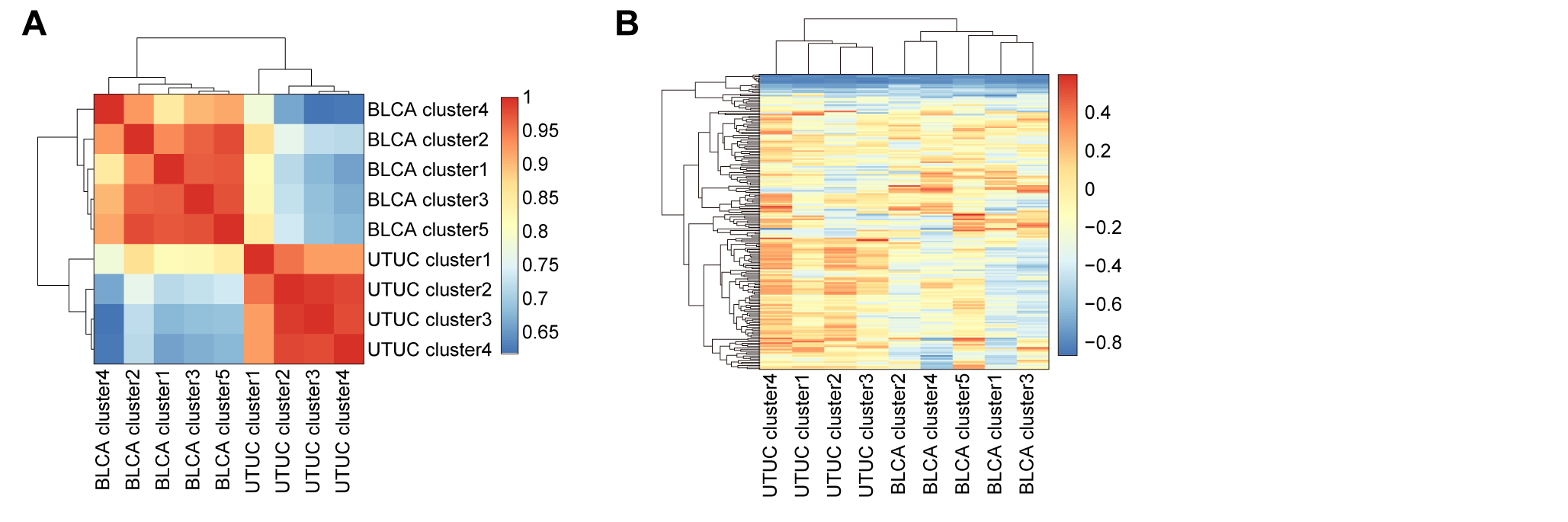

Supplement: Supplementary Figure 4 — Similarities and disparities of the tumor cell subpopulations between BLCA and UTUC. (A) Correlation analysis of the transcriptomes of tumor cell subpopulations in BLCA and UTUC. (B) GSVA of the tumor cell subpopulations in UTUC and BLCA. [file Image_4.tif]

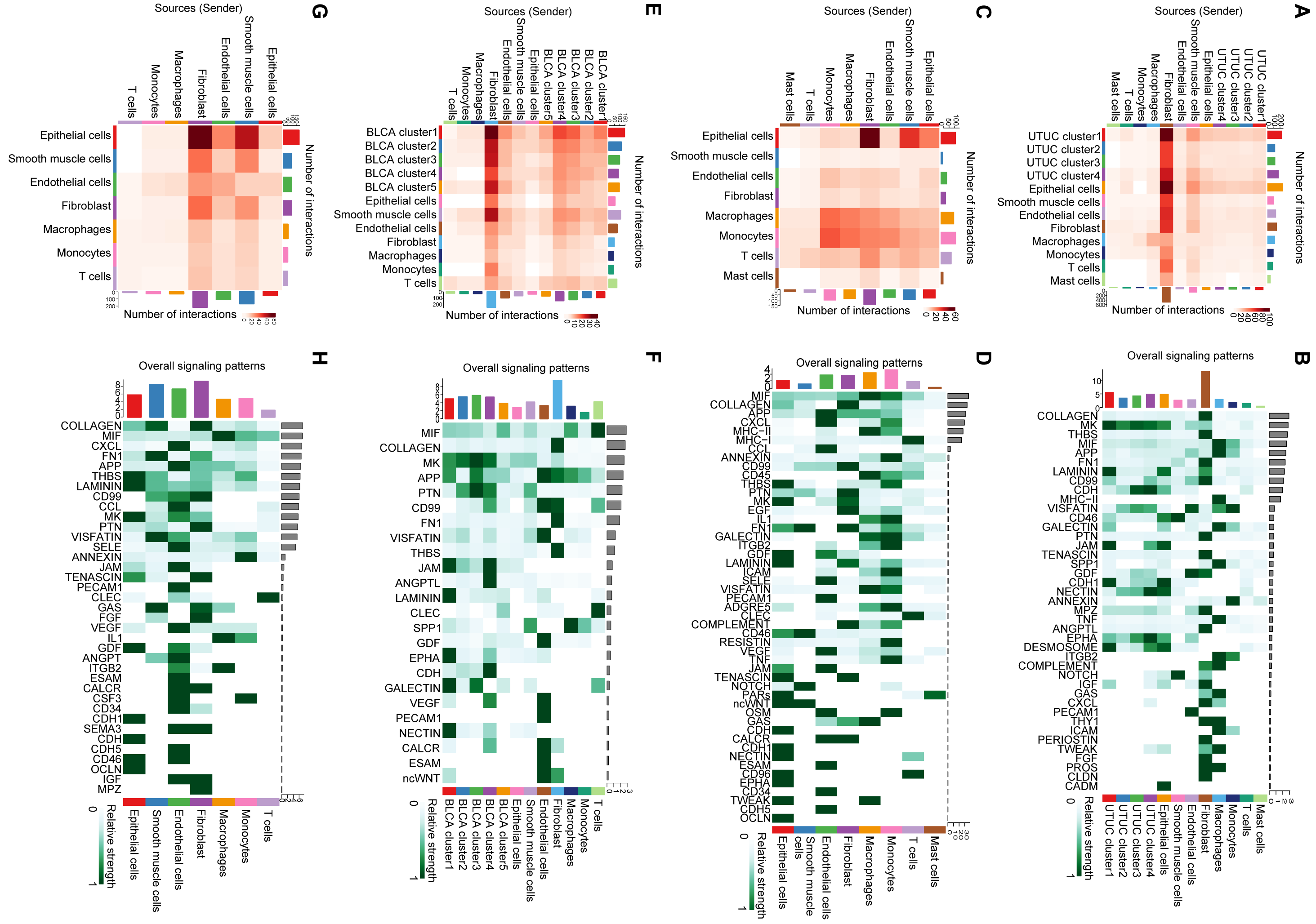

Supplement: Supplementary Figure 5 — Intercellular network construction based on the CellChat. (A, B) Intercellular network among various cell subtypes in UTUC and corresponding signaling pattern. (C, D) Intercellular network among various cell subtypes in normal ureter tissues and corresponding signaling pattern. (E-F) Intercellular network among various cell subtypes in BLCA and corresponding signaling pattern. (G, H) Intercellular network among various cell subtypes in normal bladder tissues and corresponding signaling pattern. [file Image_5.tif]

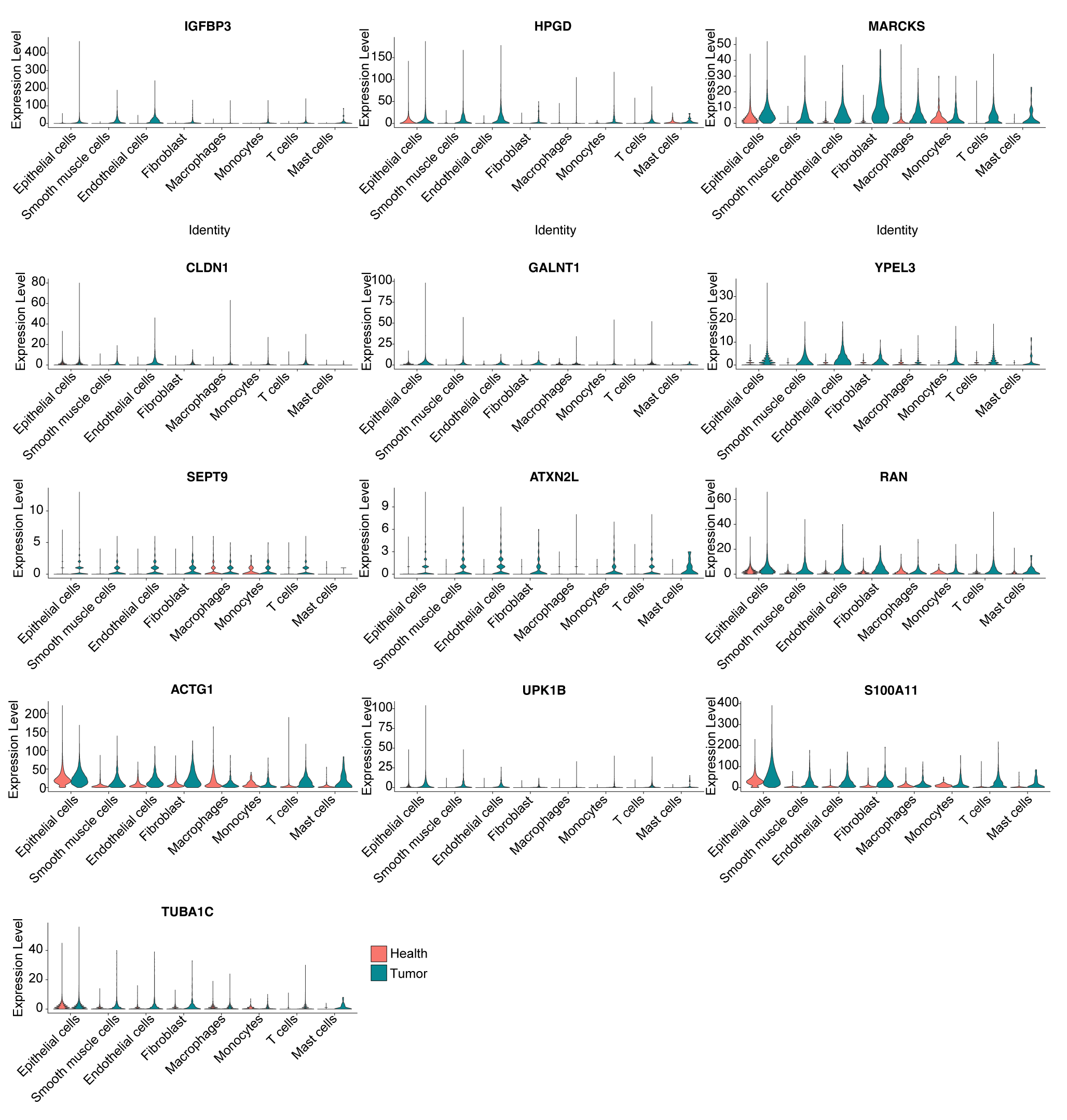

Supplement: Supplementary Figure 6 — Comparison of the relative expression pattern of 28 key DEGs closely associated with tumor cells between UTUC and normal ureter tissues. [file Image_6.tif]

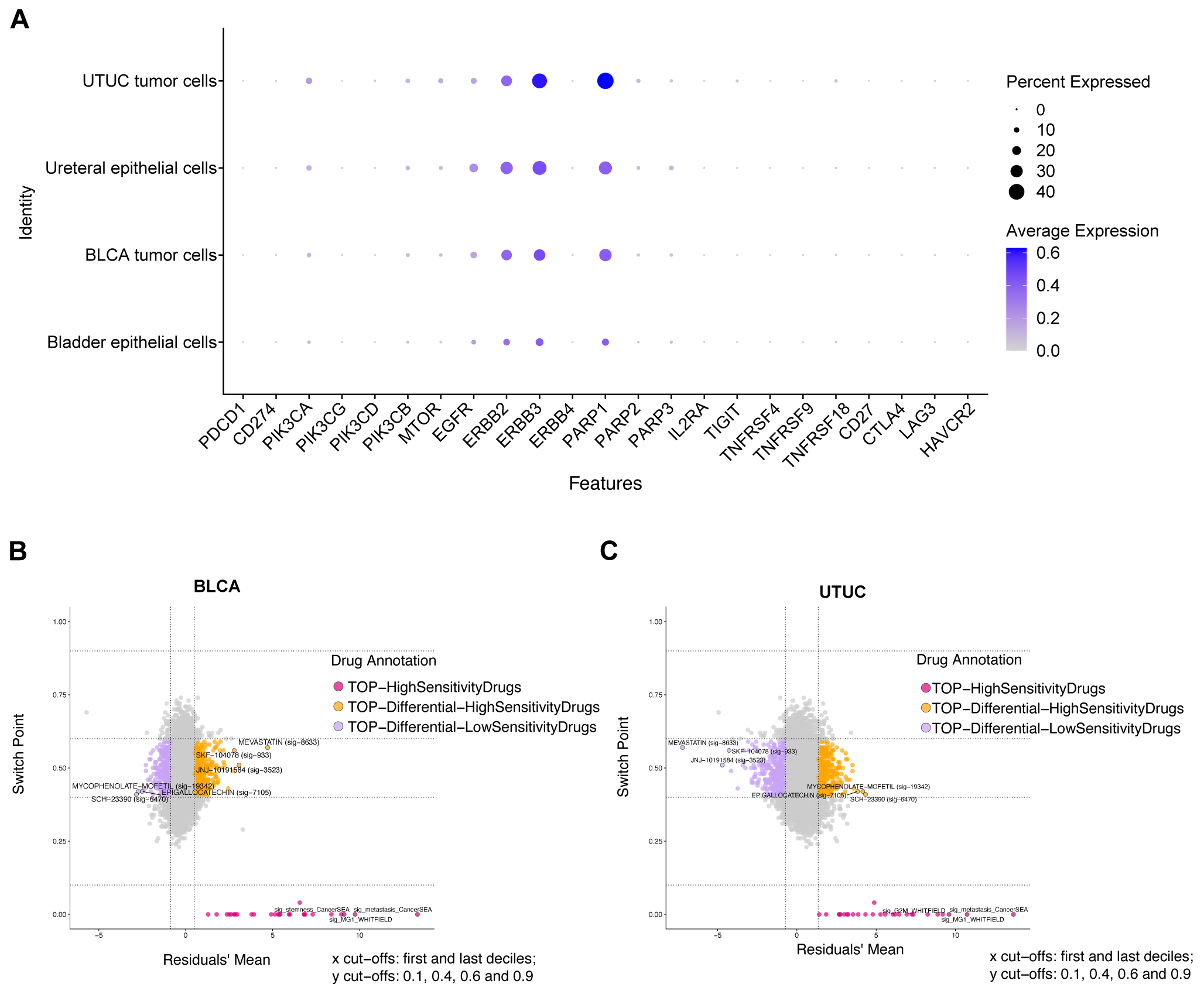

Supplement: Supplementary Figure 7 — Predictions of antitumor drug efficacy and explorations for the potential therapeutic agents. (A) Expression levels of current immunotherapeutic and targeted therapeutic targets in tumor and normal epithelial cell of UTUC and BLCA. (B) Candidate drug prediction for BLCA using R package Beyondcell. (C) Candidate drug prediction for UTUC using R package Beyondcell. [file Image_7.tif]
